# Compounds from *Sorindeia juglandifolia* (Anacardiaceae) exhibit potent anti-plasmodial activities *in vitro* and *in vivo*

**DOI:** 10.1186/1475-2875-11-382

**Published:** 2012-11-21

**Authors:** Raceline G Kamkumo, Alvine M Ngoutane, Lauve RY Tchokouaha, Patrick VT Fokou, Eugénie AK Madiesse, Jennifer Legac, Jean JB Kezetas, Bruno N Lenta, Fabrice F Boyom, Theophile Dimo, Wilfred F Mbacham, Jiri Gut, Philip J Rosenthal

**Affiliations:** 1 Laboratory of Animal Physiology, Department of Animal Biology and Physiology, Faculty of Science, University of Yaoundé 1, PO Box 812, Yaoundé, Cameroon; 2 Laboratory for Phytobiochemistry and Medicinal Plants Studies, Department of Biochemistry, Faculty of Science, University of Yaoundé 1, PO Box 812, Yaoundé, Cameroon; 3 Laboratory for Public Health Research Biotechnologies, Biotechnology Centre, University of Yaoundé 1, PO Box 8094, Yaoundé, Cameroon; 4 Department of Chemistry, Faculty of Science, University of Bamenda, Bamenda, Cameroon; 5 Department of Medicine, Division of Infectious Diseases, University of California, San Francisco, 94943, USA

**Keywords:** Malaria, Drug discovery, *Sorindeia juglandifolia*, *Plasmodium falciparum*, *Plasmodium berghei*

## Abstract

**Background:**

Discovering new lead compounds against malaria parasites is a crucial step to ensuring a sustainable global pipeline for effective anti-malarial drugs. As far as we know, no previous phytochemical or pharmacological investigations have been carried out on *Sorindeia juglandifolia.* This paper describes the results of an anti-malarial activity-driven investigation of the fruits of this Cameroonian plant.

**Methods:**

Air-dried fruits were extracted by maceration using methanol. The extract was fractionated by flash chromatography followed by column chromatography over silica gel, eluting with gradients of hexane-ethyl acetate mixtures. Resulting fractions and compounds were tested *in vitro* against the *Plasmodium falciparum* chloroquine-resistant strain W2, against field isolates of *P. falciparum*, and against the *P. falciparum* recombinant cysteine protease falcipain-2. Promising fractions were assessed for acute toxicity after oral administration in mice. One of the promising isolated compounds was assessed *in vivo* against the rodent malaria parasite *Plasmodium berghei*.

**Results:**

The main end-products of the activity-guided fractionation were 2,3,6-trihydroxy benzoic acid (1) and 2,3,6-trihydroxy methyl benzoate (2). Overall, nine fractions tested against *P. falciparum* W2 and falcipain-2 were active, with IC_50_ values of 2.3-11.6 μg/ml for W2, and 1.1-21.9 μg/ml for falcipain-2. Purified compounds (1) and (2) also showed inhibitory effects against *P. falciparum* W2 (IC50s 16.5 μM and 13.0 μM) and falcipain-2 (IC50s 35.4 and 6.1 μM). In studies of *P. falciparum* isolates from Cameroon, the plant fractions demonstrated IC_50_ values of 0.14-19.4 μg/ml and compounds (1) and (2) values of 6.3 and 36.1 μM. *In vivo* assessment of compound (1) showed activity against *P. berghei* strain B, with mean parasitaemia suppressive dose and curative dose of 44.9 mg/kg and 42.2 mg/kg, respectively. Active fractions were found to be safe in mice after oral administration of 7 g/kg body weight.

**Conclusions:**

Fractions of *Sorindeia juglandifolia* and two compounds isolated from these fractions were active against cultured malaria parasites, the *P. falciparum* protease falcipain-2, and in a rodent malaria model. These results suggest that further investigation of the anti-malarial activities of natural products from *S. juglandifolia* will be appropriate.

## Background

Despite extensive recent efforts to control malaria, it remains a major public health threat throughout the tropics. Annually, 500 million people are at risk of malaria, and about one million deaths occur, primarily from disease caused by *Plasmodium falciparum*, and mostly in pregnant women and children under five years of age. With persistent severe malarial morbidity and increasing resistance to malaria drugs, including possibly new artemisinin-based combination therapy (ACT) [1], there is a compelling need for new and improved treatments for malaria. Therefore, a vibrant drug discovery pipeline is needed to ensure the availability of new effective anti-malarials.

The nations of Sub-Saharan Africa have the greatest burden of malaria, and also vital resources, that is diversified and rich floras on which their traditional medicines depend. Considering important roles for natural products in the treatment of malaria, the screening of medicinal plants to search for novel chemical entities with activity against malaria parasites is a credible approach to discover new anti-malarial leads. In this paper, the results of investigation of the fruits of *Sorindeia juglandifolia* (Anacardiaceae) growing in Cameroon as a source of anti-malarial agents are reported.

## Methods

### Plant selection and collection

In the framework of a random study of Cameroonian plants as sources of anti-malarial agents, the fruits of *S. juglandifolia* were collected during the rainy season in Mt. Kalla, Yaoundé area, Cameroon in October, 2010. The plant sample was brought to the Laboratory for Phytobiochemistry and Medicinal Plants Studies, University of Yaoundé I, Cameroon for phytochemical and biological studies. Botanical identification was done at the National Herbarium, Yaoundé by Mr. Victor Nana, where a voucher specimen is deposited under the identification number 9176 SRFCam. The genus *Sorindeia* includes trees, shrubs or lianas that are confined to tropical Africa, including Madagascar, the Comoro Islands and the Mascarene Islands [2]. *Sorindeia juglandifolia* is a tree of up to 23 m height of which no specific use and no pharmacological studies have been found. Therefore, the approach adopted in this study differs from the reverse pharmacology approach [3], since there is no evidence of plant use against malaria. A related species, *Sorindeia mildbraedii*, is used to provide chewing sticks for oral hygiene [4].

### Plant extraction and structure elucidation

#### Reagents and materials

Melting points were determined on a Büchi-540 melting point apparatus. UV spectra were determined on a Spectronic Unicam spectrophotometer. IR spectra were determined on a JASCO Fourier Transform IR-420 spectrometer. ^1^H- and ^13^C NMR spectra were run on a Bruker spectrometer equipped with 5 mm ^1^H and ^13^C probes operating at 500 and 125 MHz, respectively, with TMS as internal standard. Silica gel 230–400 mesh (Merck) and silica gel 70–230 mesh (Merck) were used for flash and column chromatography, while precoated aluminum-backed silica gel 60 F_254_ sheets were used for TLC. Spots were visualized under UV light (254 and 365 nm) or using MeOH-H_2_SO_4_ reagent.

#### Extraction and fractionation

The ground fruits (2 kg) were extracted at room temperature with methanol (2× 5 L, 48 h each). Solvent was evaporated under reduced pressure to yield 28g of extract. A portion (20g) of the extract was fractionated by flash chromatography over silica gel (70–230 mesh, Merck, 7 × 42 cm), eluting with a gradient of increasing polarity of mixtures of hexane-ethyl acetate 100:0–0:100, resulting in the collection of 35 fractions of 500mL each, which were combined on the basis of TLC analysis to yield 18 subfractions, labelled SJFR1-SJFR18. All these fractions were tested *in vitro* against the *Plasmodium falciparum* chloroquine-resistant W2 strain and the *P. falciparum* cysteine protease falcipain-2.

Fractions SJFR8-10 [Hex:EtOAc (1:1)] and SJFR17-18 [Hex:EtOAc (0:1)] showed the best potencies against falcipain-2 and *P. falciparum* W2, respectively. Subsequent TLC analysis suggested the combination of the respective fractions into SJFRA and SJFRB, which were submitted to phytochemical investigation.

Fraction SJFRA [7g, composed of subfractions 20–22, obtained with Hex:EtOAc (1:1)] was subjected to column chromatography over silica gel (230–400 mesh, Merck, 5 × 42 cm), eluting with gradient mixtures of hexane-ethyl acetate 30:70–80:20, resulting in 82 subfractions of 200 mL, combined on the basis of TLC analysis into two subfractions, SJFRA1 (1–25) (3.8g), and SJFRA2 (26–82) (0.3g).

Subfraction SJFRA1 showed significant anti-plasmodial activity, and was, therefore, subjected to column chromatography over silica gel (230–400 mesh, Merck, 5 × 42 cm), eluting with gradient mixtures of hexane-ethyl acetate 30:70 – 20:80 to yield SJFRA1-41, identified as 2,3,6-trihydroxy benzoic acid (**1**, 125mg).

Column chromatography over silica gel (230–400 mesh, Merck, 5 × 42 cm) of SJFRB [6g, composed of subfractions 34–35, obtained with Hex:EtOAc (0:1)] eluting with gradient mixtures of hexane-ethyl acetate 80:20 – 0:100 and EtOAc- MeOH (1:0 to 98:2) yielded SJFRB-41, identified as 2,3,6-trihydroxy methyl benzoate (**2**, 35mg).

### Evaluation of biological activities

The protocol described in this report was approved by the Institutional Review Board (IRB-No: 001/UY11 BTC/ IRBI 2009), Biotechnology Centre, University of Yaoundé 1, Cameroon, and the World Health Organization Research Ethics Review Committee (WHO ERC-A80689).

### In vitro screening of *Sorindeia juglandifolia* extracts against *Plasmodium falciparum* W2

*Plasmodium falciparum* strain W2, known to be resistant to chloroquine and other anti-malarials [5], was cultured in sealed flasks at 37°C, in a 3% O_2_, 5% CO_2_ and 91% N_2_ atmosphere in RPMI 1640, 25 mM HEPES, pH 7.4, supplemented with heat inactivated 10% human serum and human erythrocytes to achieve a 2% haematocrit. Serum and erythrocytes were purified from blood voluntarily given by adults with no recent contact with malaria, and who have signed an informed consent form. Parasites were synchronized in the ring stage by serial treatment with 5% sorbitol (Sigma) [6] and tested at 1% parasitaemia. Stock solutions of plant extracts were prepared as 1 mg/ml in DMSO, diluted as needed for individual experiments, and tested in triplicate as described previously [7,8]. The stock solutions were diluted in supplemented RPMI 1640 medium so as to have at most 0.2% DMSO in the final reaction medium. Equal volumes (100μl) of inhibitors and 1% parasitaemia, 4% haematocrit culture were then added and gently mixed thoroughly. Negative controls contained equal concentrations of DMSO. Positive controls contained 1 μM chloroquine phosphate (Sigma). Cultures were incubated at 37°C for 48 h. Parasites at the ring stage were thereafter fixed by replacing the serum medium by an equal volume of 1% formaldehyde in PBS. Aliquots (5 μl) of each culture were then added to round-bottom polystyrene 96 well plates containing 150 μl of 100 mM NH4Cl, 0.1% Triton X- 100 and 1 nM YOYO-1 nuclear dye (Molecular Probes) in PBS, and the parasitaemia of treated and control cultures were compared using a Becton-Dickinson FACSort flow cytometer equipped with AMS-1 loader (Cytek Development) to count nucleated (parasitized) erythrocytes. Data acquisition was performed using Cell Quest software. These data were normalized to percent control activity and 50% inhibitory concentrations (IC_50_s ) were calculated using Prism 5.0 software (GraphPad) with data fitted by nonlinear regression to the variable slope sigmoidal dose–response formula, y = 100/[1 + 10^(logIC50− x)*H*^ where *H* is the hill coefficient or slope factor [5].

### In vitro screening of *Sorindeia juglandifolia* extracts against *Plasmodium falciparum* field isolates

#### Blood sampling and purification of Plasmodium falciparum isolates

*Plasmodium falciparum* isolates were prepared as described previously [9]. Blood was collected from outpatients presenting at the Etoug-Ebe Baptist Health Centre in Yaoundé with uncomplicated malaria. Patients older than five years of age with mono infection with *P. falciparum* and parasite density greater than 6,000 asexual parasites per microliter of blood were considered for inclusion in the study. Those with recent history of self-medication with anti-malarial drugs were excluded. After patients agreed to participate in the study and signed the consent form, 5 mL of blood was collected from them by venipuncture into heparinized tubes. The blood was centrifuged at 3,000 rpm for 15 min and the supernatant discarded. The pellets were suspended in 10 mL of pre-warmed RPMI 1640 medium and centrifuged again twice at 3,000 rpm for 5 min, and the supernatant was then discarded. The volume of the packed-cells was estimated and mixed with an equal amount of RPMI 1640, 25 mM HEPES, pH 7.4, and subsequently used at 4% haematocrit for experiments. Thin smears were evaluated to assess the parasitaemia, the developmental stages of parasites, and the colour, shape and size of red blood cells. Nine such isolates were prepared and subsequently used for experiments.

#### Evaluation of antiplasmodial activity

The activity of plant products was assessed as previously described [9]. All the fractions and pure compounds that showed activity against *P. falciparum* W2 and falcipain-2 were tested against the *P. falciparum* field isolates. Stock solutions of plant extracts were prepared at 1 mg/ml in DMSO and diluted as needed for individual experiments in 96-well plates using RPMI 1640, to achieve a maximum final DMSO concentration of 0.2%. Chloroquine was used as positive control at 1 μM, and the negative control was an equal concentration of DMSO. To assess the susceptibility of parasites to drugs, they were maintained in RPMI 1640 supplemented with 25 mM HEPES buffer (pH 7.4), 0.05% ampicillin, and 10% AB+ human serum at 37°C, in a candle jar. To achieve this, 200 μl parasite suspensions at 4% haematocrit and 0.1%– 0.3% parasitaemia were added to 50 μl of each concentration of inhibitor in individual wells, each tested in triplicate. Cultures were incubated at 37°C for 48 h in a candle jar. To estimate the effects of inhibitors after 48 h, thin smears from treated and control cultures were fixed in methanol for 30 s, stained using fresh 5% Giemsa solution (Sigma) in phosphate buffer (pH 7.1), and examined by counting the parasitaemia per 100 erythrocytes. IC_50_s were calculated from curves plotting percent growth versus drug concentration using Prism 5.0 software with data fitted by nonlinear regression to the variable slope sigmoidal dose–response formula y = 100/[1 + 10^(logIC50− x)*H*^, where *H* is the Hill coefficient or slope factor [5].

### Assessment of falcipain-2 inhibition by plant extracts

Falcipain-2 activity was measured as hydrolysis of the fluorogenic substrate benzyloxycarbonyl-Leu-Arg-7-amino-4-methyl-coumarin (Z-Leu-Arg-AMC) using a Fluoroskan II spectrofluorometer, as described previously [10]. For inhibitor assays, recombinant falcipain-2 prepared as described previously [11] was incubated with inhibitors (added from stocks concentrated 100- to 1,000-fold in dimethyl sulfoxide [DMSO]) with 10 mM dithiothreitol, pH 5.5, for 30 min at room temperature before the substrate (50 μM) was added. Equal concentrations of enzyme were used for each experiment. Multiple inhibitor concentrations were studied in triplicate, and the rates of hydrolysis were determined and compared to those of controls containing equal concentrations of DMSO. All values were normalized to the control activity, and IC_50_s were calculated with the Prism 5.0 program, with data fitted by nonlinear regression.

### In vivo assessment of 2,3,6-trihydroxy benzoic acid against *Plasmodium berghei*

Compound **1** (2,3,6-trihydroxy benzoic acid), obtained from the fractionation of *S.juglandifolia* extract, was assessed *in vivo* using the *Plasmodium berghei* rodent parasite model, according to the procedure described by Okokon *et al.*[12] with a few modifications. *Plasmodium berghei* strain B (MRA-406, MR4, ATCC® Manassas Virginia) was obtained from BEI Resources [13] and used for the experiments. Female Swiss albino mice aged eight weeks and weighing ~20 g were used for efficacy and toxicity experiments. These animals were bred in the animal house of the Faculty of Medicine, University of Yaoundé I. They were fed a standard laboratory diet (S.P.C. Ltd., Bafoussam, Cameroon) and given clean tap water *ad libitum*. Animal welfare requirements were strictly considered during the experiments, as described in the protocol approved by the National Ethics Committee (Reg. No.FWAIRB00001954). To start infections, a cryotube of parasites was thawed and the undiluted contents were injected immediately i.p. into naïve recipient mice. Three days after infection, the parasitaemia was evaluated daily in tail vein blood through Giemsa-stained thin smear observation under oil immersion at 100X magnification. Passage of infection into new mice consisted of an i.p. injection of 200 μL of 10^6^ infected erythrocytes/mL sterile PBS. The test compound was dissolved and diluted in Tween 20: Ethanol (7:3 v/v).

### Evaluation of the suppressive effect of the compound

Preliminary screening of the compound consisted of a suppressive test. To achieve this, animals were infected i.p. with 200 μL of 10^6^ infected erythrocytes/mL sterile PBS. Four hours after infection, duplicate batches of animals were treated as follows: four batches of six mice each were given test drug orally at respective dosages of 10, 30, 50 and 100 mg/kg, and subsequently once daily for four consecutive days. Negative controls consisted of one batch of 6 infected mice receiving no drug (dose 0), and one batch of six uninfected animals. Positive controls consisted of one batch of infected mice receiving quinine sulfate at 24 mg/kg.

### Evaluation of the curative effect of the compound

Curative assays were subsequently conducted using the same setting, with animal receiving drug at daily oral dosages of 10, 30, 50 and 100 mg/kg for 5 consecutive days, starting day 3 (72 hours) after infection. Thin blood smears were made daily from the tail blood of mice and the level of parasitaemia assessed by examination of Giemsa-stained smears using a microscope at 100X magnification. In both cases, activity was expressed as ED_50_s that were calculated based on percent parasitaemia obtained 24 h after the last drug administration using GraphPad Prism 5.0 software.

### Evaluation of the acute toxicity of active extracts

The acute toxicity profile of the active fractions was determined as previously described [6,8] and according to the Organization for Economic Cooperation and Development (OECD) protocol briefly described below, with some modifications [14]. Before the experiments, animals were starved for 12 h in wire mesh bottom cages to prevent coprophagy but allowed free access to water.

Mice were studied in groups of 6 animals (3 males and 3 females). They received the extract solutions in olive oil *per os* in doses ranging from 1,000 to 7,000 mg/kg. The negative control group received 1ml olive oil. The animals were carefully observed for 2 h, and thereafter for 7 days during which mortality, body weights and gross behavioural changes were recorded daily.

## Results and discussion

### Phytochemical investigation of the fruit extract of *Sorindeia juglandifolia*

**Figure 1:**
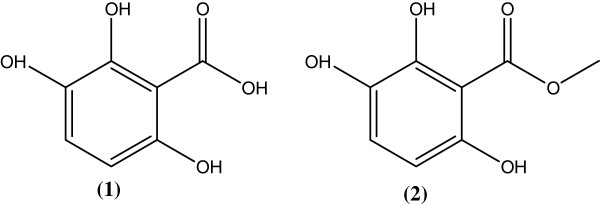
**Antiplasmodial compounds from the fruits of *Sorindeia juglandifolia *(Anacardiaceae).** Compounds **1** and **2** were isolated from the fruits of *Sorindeia juglandifolia* and characterized by means of physico-chemical and spectroscopic techniques and identified as 2,3,6-trihydroxy benzoic acid (**1**) and 2,3,6-trihydroxy methyl benzoate (**2**).

The air-dried and ground fruits of *S. juglandifolia* were extracted at room temperature using MeOH. The extract was concentrated to dryness under vacuum and the residue subjected to flash chromatography. Repeated column chromatography of pooled fractions yielded two known compounds, 2,3,6-trihydroxy benzoic acid (**1**), m/z= 170; mp= 188.5-190; yellow crystals and 2,3,6-trihydroxy methyl benzoate (**2**), m/z= 184; mp= 139–140.5; pale yellow crystals with respective purification yields of 0.062% and 0.0017% relative to the weight of the starting plant material (Figure 1). These two phenol derivatives have not generally been obtained from natural sources, but related compounds, such as gallic acid and derivatives, are ubiquitous in the plant kingdom and readily available from natural sources. In addition to these pure compounds, fractions were afforded and were also tested for anti-plasmodial activity.

### *In vitro* activity of *Sorindeia juglandifolia* extracts against *Plasmodium falciparum* W2 and recombinant falcipain-2

Results achieved from the *in vitro* assessment of plant extracts are presented in Table 1. From these results, six fractions (SJFR1, SJFR2, SJFR8, SJFR9, SJFR10, SJFR17,) and seven fractions (SJFR1, SJFR2, SJFR4, SJFR9, SJFR15, SJFR17, SJFR18) out of the 18 afforded through flash chromatography exerted inhibitory effects against *P. falciparum* W2 strain and recombinant falcipain-2, respectively. The IC_50_ values were 2.3-11.6 μg/ml for W2, and 1.1-21.9 μg/ml for falcipain-2. These activities support further investigation of the fractions to identify the active principles.

**Table 1:** **Inhibition of *Plasmodium falciparum *W2 and recombinant falcipain-2 by *Sorindeia juglandifolia *extracts**

**Fractions /Compounds**	**Solvent system**	***P. falciparum*W2- IC**_**50**_**±SD (μg/ml)**	**Falcipain-2 IC**_**50**_**±SD (μg/ml)**
SJFR1		**6.24**±1.01	**8.22**±1.09
SJFR2	Hex:EtOAc 10%	**11.55**±2.45	**6.02**±3.81
SJFR3		**>10**	**>10**
SJFR4		**>10**	**21.90** ±1.85
SJFR5	Hex:EtOAc 25%	**>10**	**>10**
SJFR6		**>10**	**>10**
SJFR7		**>10**	**>10**
SJFR8		**2.76**±0.08	**>10**
SJFR9		**2.30**±0.97	**5.42**±0.65
SJFR10	Hex:EtOAc 50/50	**2.46**±0.29	**>10**
SJFR11		**>10**	**>10**
SJFR12		**>10**	**>10**
SJFR13		**>10**	**>10**
SJFR14		**>10**	**>10**
SJFR15	Hex:EtOAc 75%	**>10**	**17.31**±2.51
SJFR16		**>10**	**>10**
SJFR17		**8.47**±0.41	**1.12**±0.16
SJFR18	EtOAc 100%	**>10**	**1.14**±0.10
CQ		0.05±0.01	
ART		0.007±0.01	
E64		ND	0.049±0.003
2,3,6-trihydroxy benzoic acid (**1**)		16.47±0.47 μM	35.41±22.37μM
2,3,6-trihydroxy methyl benzoate (**2**)		13.04±1.63 μM	6.09±0.87 μM

IC_50_= Concentration that killed/inhibited 50% of parasites/enzyme relative to negative control. S.D. = standard deviation, the drugs were tested in triplicate. Positive controls were CQ = chloroquine, ART= artemisinin, and E-64 = l-transepoxy-succinyl-leucylamido-(4-guanidino)-butane.

Detailed investigation of promising fractions afforded 2,3,6-trihydroxy benzoic acid (**1**) and 2,3,6-trihydroxy methyl benzoate (**2**), which also showed inhibitory effects in the low micromolar range, with IC_50_ values of 16.5 and 13.0 μM against *Plasmodium falciparum* W2, and 35.4 and 6.1 μM against falcipain-2. The potency exerted by the purified compounds confirms the preliminary results obtained with fractions, suggesting potent anti-malarial activity of natural products from *S. juglandifolia*. Further studies are required to identify the most potent active compounds from *S. juglandifolia* and to determine the biochemical targets of these compounds.

### *In vitro* activity of *Sorindeia juglandifolia* extracts against field strains of *Plasmodium falciparum*

The nine fractions that showed potency against *P. falciparum* W2 strain and falcipain-2 (SJFR1, SJFR2, SJFR4, SJFR8, SJFR9, SJFR10, SJFR15, SJFR17, and SJFR18) were tested *in vitro* against fresh *P. falciparum* isolates (Additional file 1). The results showed potent effects of plant fractions against *P. falciparum* isolates. However, results varied greatly from isolate to isolate, with IC_50_ values of 0.14-19.4 μg/ml. All the fractions showed potency against at least one *P. falciparum* isolate. Isolate E01 was the most sensitive to all the fractions, with IC_50_ values below 1 μg/ml. Overall, SJFR10, SJFR17, and SJFR18 inhibited all the nine isolates with IC_50_ ranging from 0.20-16.44 μg/ml. The positive control (CQ) was active against all the isolates, with IC_50_s from 16.6-38.4 ng/ml. Compounds (**1**) and (**2**) also showed activity against all nine isolates, with IC_50_ values from 6.3- 36.1 μM.

Of note, the fresh *P. falciparum* isolates were 10–20 fold more sensitive to the tested fractions, compared to the results obtained with the same fractions against the W2 reference strain. Similar findings were recently described, with variability of results in the assessment of the anti-plasmodial activity of Cameroonian Annonaceae plant extracts [9]. Overall, no significant changes were observed in colour, shape and size of red blood cells of treated mice relative to negative controls.

### *In vivo* activity of 2,3,6-trihydroxy benzoic acid against *Plasmodium berghei* B

**Figure 2:**
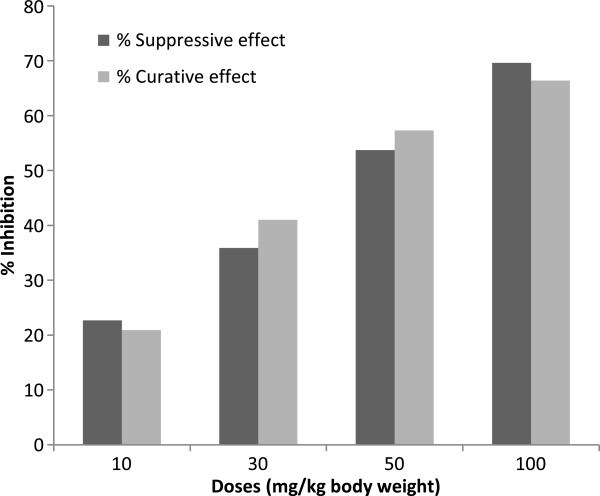
***In vivo *inhibitory effect of 2,3,6-trihydroxy benzoic acid (1) against rodent malaria.** Compound 2,3,6-trihydroxy benzoic acid (**1**) was assessed for anti-plasmodial activity *in vivo.* Suppressive and curative tests showed 50% suppressive and effective doses of 44.9 and 42.2 mg/kg, respectively.

Compound **1**, (2,3,6-trihydroxy benzoic acid), which showed good potency *in vitro* against *P. falciparum* W2 and against falcipain-2 was assessed *in vivo*. *In vitro* results were confirmed through assays using *P. berghei* strain B that showed modest activity at doses ranging from 10–100 mg/kg body weight (Table 2). When compared to *in vitro* potency, this moderate activity of compound (**1**) *in vivo* may be due to many parameters such as bioavailability and metabolism that vary. Detailed studies that include compound optimization, *in vitro* and *in vivo* screening, and cytotoxicity study will indicate further directions. Compound (**1**) exerted a dose-dependent suppression of parasites when evaluated after 6 days (Figure 2). Similarly, parasite growth appeared to be inhibited in a dose-dependent manner during the 5-day curative test (Figure 2). In these experiments the 50% suppressive dose and effective doses were 44.9 ± 1.6 mg/kg and 42.2 ± 1.6 mg/kg, respectively.

**Table 2:** ***In vivo *anti-malarial activity of 2,3,6-trihydroxy benzoic acid (1)**

**Drug dose (mg/kg)**	**Mean % Suppression/Inhibition**	
*Suppressive test*		
10	22.73	
30	35.95	^*c*^SD_50_ = 44.9 ± 1.6 mg/kg
50	53.66	
100	69.63	
Quinine sulfate (24 mg/kg)	71.34	
*Curative test*		
10	20.95	
30	41.00	^*c*^ED_50_= 42.2 ± 1.6 mg/kg
50	57.27	
100	66.40	
Quinine sulfate (24 mg/kg)	89.50	

^*c*^Suppressive/Effective dose at 50%= concentration of drug that cleared/reduced parasitaemia by 50%. Percentages of inhibition were calculated based on the parasitaemia obtained 24 h after the last drug administration.

### Toxicity profile of *Sorindeia juglandifolia* extracts

Promising extracts were assessed for acute toxicity through oral administration in mice at doses of 1,000, 3,000, 5,000 and 7,000 mg/kg. Apart from transient prostration and loss of appetite at doses of 5,000 and 7,000 mg/kg, no major behavioral changes among experimental animals were observed, and no deaths were seen at doses ≤ 7,000 mg/kg body weight. This indicates that LD_50_ values of tested fractions are > 7,000 mg/kg, 70–140 folds above doses with anti-plasmodial activity. According to the OECD guidelines [14-16], these results suggest that the fractions are safe.

## Conclusions

*Sorindeia juglandifolia* fruit extracts showed promising anti-plasmodial properties in *in vitro* and *in vivo* experiments. The present study also identified two active principles: 2,3,6-trihydroxy benzoic acid and 2,3,6-trihydroxy methyl benzoate with anti-malarial activity. These results support further investigation of natural products from *S. juglandifolia* for anti-malarial drug development. Within this framework, further studies will focus on chemical features of active principles from other promising fractions, impact of fruit collection time on quality, quantity, and activity of isolated metabolites, and the eventual reversal effect of active natural products on parasites resistance to chloroquine in a clinical setting as previously experimented by Willcox and Rasoanaivo [17].

## Abbreviations

OECD: Organization for Economic Cooperation and Development; ACT: Artemisinin-based combination therapies; WHO: World Health Organization; CQ: Chloroquine; DMSO: Dimethyl sulfoxide; NMR: Nuclear magnetic resonance; UV: Ultra violet; IR: Infra-red; TLC: Thin layer chromatography; UCSF-SFGH: University of California San Francisco-San Francisco general hospital; ip: Intra-peritoneal.

## Competing interests

The authors declare that they have no competing interests.

## Authors’ contributions

FFB and RGK participated in plant selection and collection, and analyzed botanical/ethnomedicinal data in collaboration with the ethnobotanist. RGK, AMN, LRTY, PVTF, EAKM carried out the *in vitro* (on isolates) and *in vivo* anti-plasmodial assays that were conceived, designed and coordinated by FFB and WFM. They also contributed to data analysis and manuscript drafting. JJB and BNL carried out the phytochemical study of the plant sample and structure elucidation. TD designed and coordinated the toxicity studies and contributed to the manuscript drafting. JL and JG carried out the *in vitro* assays against *Plasmodium falciparum* W2 and falcipain 2 and also contributed to manuscript drafting. These studies were conceived and coordinated by PJR who also critically improved the quality of the manuscript. All the authors read and approved the final version of manuscript.

## Supplementary Material

Additional file 1 **Inhibition of *P. falciparum *isolates by *Sorindeia juglandifolia *fractions and purified compounds.** Click here for file
