# Supplementary material for: Compounds from Sorindeia juglandifolia (Anacardiaceae) exhibit potent anti-plasmodial activities in vitro and in vivo
Source: Malar J. 2012 Nov 21;11:382. doi: 10.1186/1475-2875-11-382 (PMC3519527; doi:10.1186/1475-2875-11-382)
Supplement: Additional file 1 — Inhibition of P. falciparum isolates by Sorindeia juglandifolia fractions and purified compounds. [file 1475-2875-11-382-S1.docx]

Additional file 1 Inhibition of *P. falciparum* isolates by *Sorindeia juglandifolia* fractions and purified compounds

| **Code of Inhibitor** | | CQ (ng/ml) | SJFR1 | SJFR2 | SJFR4 | SJFR8 | SJFR9 | SJFR10 | SJFR15 | SJFR17 | SJFR18 | CPD1 (µM) | CPD2 (µM) |
| --- | --- | --- | --- | --- | --- | --- | --- | --- | --- | --- | --- | --- | --- |
| **Isolates/ *^a^*IC_50_±SD of plant extracts (µg/ml)** | E01 | 25.42±3.47 | **0.43±0.11** | **0.19±0.08** | **0.43±0.31** | **0.14±0.26** | **0.23±0.39** | **0.71±0.07** | **0.27±0.07** | **0.21±0.32** | **0.20±0.08** | 11.32±0.92 | 7.02±0.07 |
|  | E02 | 23.33±19 | 7.69±0.49 | 12.92±0.63 | 1.03±0.34 | **0.22±0.34** | **0.44±0.15** | 1.96±0.97 | 1.02±0.67 | **0.23±0.12** | **0.21±0.12** | 17.09±0.48 | 14.68±0.15 |
|  | E03 | 38.39±4.37 | **0.95±0.31** | 4.40±1.21 | 10.83±3.26 | 4.92±0.97 | >20 | 4.71±1.59 | **0.90±0.21** | 1.09±0.55 | 7.10±4.33 | 21.97±0.93 | 19.76±0.38 |
|  | E04 | 27.22±0.72 | **0.54±0.52** | 14.02±0.52 | > 20 | > 20 | **0.22±0.08** | 1.16±0.25 | > 20 | **0.90±0.55** | 2.14±0.12 | 24.82±1.04 | 18.82±1.04 |
|  | E05 | 16.63±1.48 | 12.21±0.28 | > 20 | > 20 | 1.52±0.62 | 2.22±0.18 | 3.06±0.35 | > 20 | 4.13±0.24 | 1.90±0.56 | 6.32±0.16 | 12.20±0.02 |
|  | E06 | 19.21±4.86 | > 20 | 11.06±0.57 | > 20 | 2.56±0.58 | 12.62±2.21 | 9.17±0.55 | > 20 | 9.32±1.23 | 8.32±0.23 | 36.05±2.01 | 23.10±2.01 |
|  | E07 | 24.33±3.32 | > 20 | > 20 | 13.43±3.78 | 3.65±0.67 | 1.91±1.04 | 2.10±0.43 | 19.42±2.27 | 11.36±0.69 | 6.64±0.73 | 33.67±0.61 | 26.69±0.02 |
|  | E08 | 18.67±0.94 | > 20 | > 20 | 12.46±1.62 | 6.11±1.74 | >20 | 0.98±0.41 | >20 | 16.44±1.92 | 0.64±0.46 | 9.92±4.82 | 13.28±1.85 |
|  | E09 | 18.78±1.87 | 8.50±0.27 | > 20 | > 20 | 3.16±0.64 | >20 | 2.42±0.76 | >20 | 4.42±0.28 | 6.34±0.88 | 19.77±0.14 | 24.40±0.05 |

The susceptibility of *Plasmodium falciparum* isolates to plant fractions was evaluated in culture; ***^a^***IC_50_ = Concentration of extract that inhibited 50% of parasites, relative to negative control. S.D. = standard deviation, the drugs were tested in triplicate. En = Etoug Ebe isolate number. Positive controls was CQ = Chloroquine.

CPD1,2 = Compounds 1,2. Bold=very good activity
